# Regulation of hippocampal synaptic plasticity thresholds and changes in exploratory and learning behavior in dominant negative NPR-B mutant rats

**DOI:** 10.3389/fnmol.2014.00095

**Published:** 2014-12-01

**Authors:** Gleb Barmashenko, Jens Buttgereit, Neil Herring, Michael Bader, Cemil Özcelik, Denise Manahan-Vaughan, Karl H. Braunewell

**Affiliations:** ^1^Guest Group, In vitro-Electrophysiology Laboratory, Department of Neurophysiology, Medical Faculty, Ruhr University BochumBochum, Germany; ^2^Department of Neurophysiology, Medical Faculty, Ruhr University BochumBochum, Germany; ^3^Experimental and Clinical Research Center, Max Delbrück Center for Molecular Medicine, Charité Medical FacultyBerlin, Germany; ^4^Max Delbrück Center for Molecular MedicineBerlin, Germany; ^5^Department of Physiology, Anatomy and Genetics, Burdon Sanderson Cardiac Science Centre – BHF Centre of Research Excellence, University of OxfordOxford, UK

**Keywords:** cGMP, exploratory, hippocampus, LTP, LTD, metaplasticity, NPR-B, memory

## Abstract

The second messenger cyclic GMP affects synaptic transmission and modulates synaptic plasticity and certain types of learning and memory processes. The impact of the natriuretic peptide receptor B (NPR-B) and its ligand C-type natriuretic peptide (CNP), one of several cGMP producing signaling systems, on hippocampal synaptic plasticity and learning is, however, less well understood. We have previously shown that the NPR-B ligand CNP increases the magnitude of long-term depression (LTD) in hippocampal area CA1, while reducing the induction of long-term potentiation (LTP). We have extended this line of research to show that bidirectional plasticity is affected in the opposite way in rats expressing a dominant-negative mutant of NPR-B (NSE-NPR-BΔKC) lacking the intracellular guanylyl cyclase domain under control of a promoter for neuron-specific enolase. The brain cells of these transgenic rats express functional dimers of the NPR-B receptor containing the dominant-negative NPR-BΔKC mutant, and therefore show decreased CNP-stimulated cGMP-production in brain membranes. The NPR-B transgenic rats display enhanced LTP but reduced LTD in hippocampal slices. When the frequency-dependence of synaptic modification to afferent stimulation in the range of 1–100 Hz was assessed in transgenic rats, the threshold for both, LTP and LTD induction, was shifted to lower frequencies. In parallel, NPR-BΔKC rats exhibited an enhancement in exploratory and learning behavior. These results indicate that bidirectional plasticity and learning and memory mechanism are affected in transgenic rats expressing a dominant-negative mutant of NPR-B. Our data substantiate the hypothesis that NPR-B-dependent cGMP signaling has a modulatory role for synaptic information storage and learning.

## INTRODUCTION

The natriuretic peptides, ANP, BNP, and CNP (A-, B-, and C- type natriuretic peptide) and their receptors, the natriuretic peptide receptors (NPRs) are widely distributed in the central nervous system (CNS). They constitute a peptide hormone-receptor signaling system with a variety of potential roles in modulating physiological brain functions (Potter et al., 2009; Potter, 2011). Whereas ANP and BNP activate the type I transmembrane guanylyl cyclase receptor, natriuretic peptide receptor-A (NPR-A), CNP activates a related cyclase, the natriuretic peptide receptor-B (NPR-B), leading to the production of the second messenger cGMP (Potter et al., 2009). NPR-A and -B display several functional domains, including an extracellular ligand-binding domain, a transmembrane domain, and an intracellular domain, which consists of a kinase homology domain (KHD), a hinge region, and a guanylyl cyclase domain catalyzing the conversion of Mg-GTP into cGMP. A ligand-dependent receptor homodimerization is essential for the enzymatic activity of the guanylyl cyclase domain (Potter, 2011).

Natriuretic peptide receptor-B leads to various physiological effects ranging from bone growth (Bartels et al., 2004; Potter et al., 2009; Potter, 2011) to CNS effects, such as axonal sprouting in dorsal root ganglion neurons (Schmidt et al., 2007; Potter et al., 2009), effects on synaptic plasticity (Decker et al., 2008, 2009, 2010), and anxiogenic effects in rats and humans (Jahn et al., 2001; Kellner et al., 2003). CNP and its receptor NPR-B are widely expressed in the rat brain including expression in the limbic system, particularly in the hippocampal regions CA1-3 (Langub et al., 1995; Herman et al., 1996). In the hippocampus NPR-B is expressed in pyramidal cells and GAD (65/67)-immunopositive interneurons. Sub-cellular expression of NPR-B can be demonstrated in neuronal process in primary hippocampal cell cultures (Brackmann et al., 2005). The second messenger cGMP and cGMP signaling-cascades are implicated in the modulation of long term potentiation (LTP), long-term depression (LTD) as well as other forms of synaptic plasticity, such as short-term plasticity (Schuman and Madison, 1991; Arancio et al., 1996; Son et al., 1998). The effect of cGMP on synaptic plasticity has been attributed to the activity of soluble guanylyl cyclases (sGCs; Arancio et al., 1996). Moreover, downstream cGMP targets such as cyclic nucleotide-gated channels and cGMP-dependent kinases (cGK or protein kinase G, PKG) contribute to different forms of synaptic plasticity (Klyachko et al., 2001; Kleppisch et al., 2003; Liu et al., 2003). More recently, we investigated whether activation of another cGMP-signaling system, the membrane guanylyl cyclase NPR-B, via activation of by its ligand CNP has an effect on hippocampal synaptic plasticity (Decker et al., 2008, 2009, 2010). When LTD and LTP stimulation was applied in area CA1 at 1 and 5 Hz and 30–100 Hz, respectively, CNP increased the magnitude of LTD while LTP induction was reduced. Thus, in the presence of CNP the threshold for LTP induction was shifted to higher stimulus frequencies (Decker et al., 2010). C-type natriuretic peptide (CNP) also decreased hippocampal network oscillations in adult rats *in vitro*, which are believed to be involved in storage of information and memory consolidation *in vivo* (Decker et al., 2009). In line with these results earlier work showed that direct application of CNP into the lateral brain ventricles affects the performance of rats in a passive avoidance learning paradigm (Telegdy et al., 1999, 2000).

Knock out of the gene for NPR-B in mice causes severe dwarfism, and the NPR-B knock out mice display seizures, female sterility, and priapism and are therefore not suitable for detailed cardiovascular or neurophysiological phenotyping (Tamura et al., 2004). To overcome this limitation, transgenic rats with ubiquitous overexpression of a dominant-negative NPR-B mutant, lacking the cytoplasmic domain (CMV-NPR-BΔKC) were generated (Langenickel et al., 2006). Receptor homodimerization of NPR-B, leading to a tight contact between the guanylyl cyclase domains, is essential for enzymatic activity. Interestingly, Tamura and Garbers (2003) have described that different NPR-B isoforms resulting from alternative splicing of the primary transcript are present in mouse tissues. In addition to full-length GC-B1, GC-B2 contains a 25 aa deletion in the KHD, and GC-B3 only retains a part of the extracellular ligand-binding domain. When GC-B2 or GC-B3 is expressed coincident with GC-B1, they act as dominant negative isoforms by virtue of blocking formation of active GC-B1 homodimers (Tamura and Garbers, 2003). It appears that these splice variants serve as dominant negative regulators of full-length GC-B. Similarly, overexpression of NPR-BΔKC leads to functional down-regulation of NPR-B signaling as indicated by blunted CNP-induced cGMP production (Langenickel et al., 2006).

In order to test whether cGMP signaling via neuronal NPR-B affects bidirectional synaptic plasticity in the rat hippocampus, and simultaneously learning and memory, we have generated dominant-negative NPR-B mutant under the control of a neuron-specific promoter (NSE-NPR-BΔKC) to specifically inhibit NPR-B signaling in the brain. After confirming the expression of the NPR-BΔKC mutant and reduction of CNP-dependent cGMP generation in brain membranes we have analyzed bidirectional plasticity *in vitro* over the range of 1–100 Hz stimulation in the hippocampal CA1 region. To further evaluate these animals in behavioral experiments we have performed open field, novel object recognition and spatial object recognition (SOR) tests. Our results support the hypothesis that the cGMP-signaling cascade linked to NPR-B and its ligand CNP play an important role in the brain, and in the hippocampus in particular, to modulate bidirectional synaptic plasticity and learning behavior.

## MATERIALS AND METHODS

### GENERATION OF TRANSGENIC RATS WITH SPECIFIC INHIBITION OF NPR-B SIGNALING IN NEURONS

**FIGURE 1:**
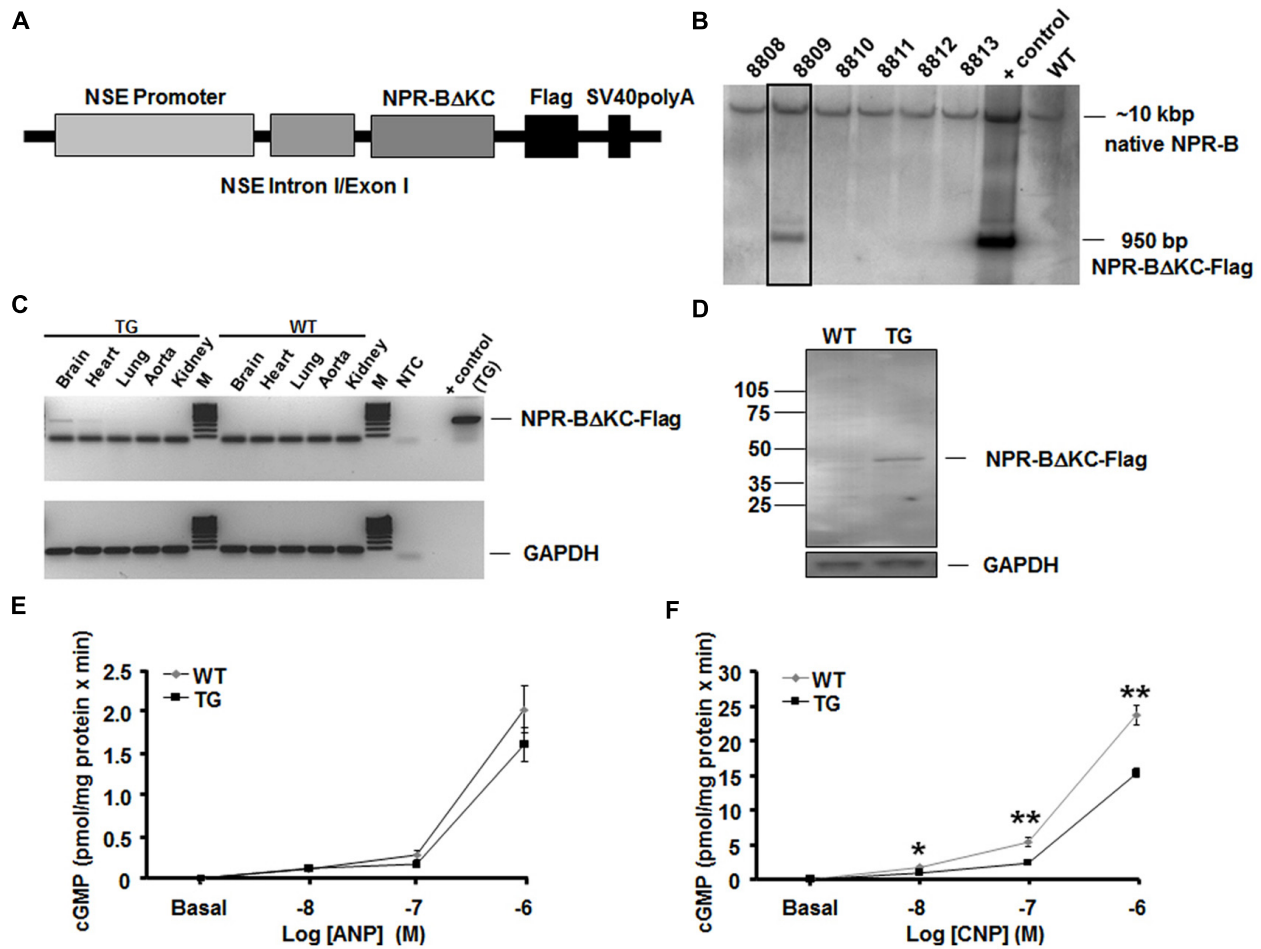
**Generation of transgenic rats. (A)** Structure of NSE-NPR-BΔKC transgenic construct using the neuron-specific enolase promoter (NSE) for neuron-specific expression. The flag/tag was used for immunodetection of the expressed protein. **(B)** Detection of a founder animal (8809, boxed lane) by Southern blotting. Native NPR-B is present in wild type animals (WT), and native NPR-B and transgene are present in the positive control and founder animal 8809. The transgene construct has a size of 950 bp, and native NPR-B of approximately 10 kbp. **(C)** Specific expression of NPR-BΔKC-Flag was demonstrated by RT-PCR. Negative control (NTC) and positive control (control + transgene) are shown for the RT-PCR showing NPR-BΔKC-Flag expression. PCR for GAPDH expression was used as loading control. M indicates a 100 bp DNA fragment ladder. **(D)** Specific expression of NPR-BΔKC-Flag as shown by Western blotting using an anti-flag antibody using extracts from brain, heart, lung, aorta, and kidney. GAPDH expression is shown as a loading control. **(E,F)** NPR-BΔKC overexpression significantly reduced CNP-dependent, but not ANP-dependent cGMP production in brain membrane preparations in transgene animals (TG) compared to wild-type animals (WT). *p < 0.05, **p < 0.01 vs. wild-type (*n* = 6 per group).

Transgenic rats overexpressing a dominant-negative NPR-B mutant under the control of a neuron-specific promoter (NSE-NPR-BΔKC; **Figure 1A**) were generated and identified by Southern blotting (**Figure 1B**) essentially as previously described (Langenickel et al., 2006). After breeding rats to homozygosity, tissue-specific expression of NSE-NPR-BΔKC in the brain was verified by reverse transcription (RT)-PCR and Western blotting. RT-PCR, Western blotting and measuring of cGMP levels in brain membrane preparation was performed as previously described (Langenickel et al., 2006; Buttgereit et al., 2010). Primary anti-flag antibodies (Life Technologies, Carlsbad, CA, USA) in combination with secondary anti-rabbit-HRP antibodies were used for detection of NSE-NPR-BΔKC-flag in Western blots. For RT-PCR the following primer pairs were used: NSE-NPR-BΔKC forward: GACAGAGAGACTGATTTCGTCC, reverse: TCACTTGTCGTCATCGTCTTTG: GAPDH: forward: CCATGGAGAAGGCTGGGG, reverse: CAAAGTTGTCATGGATGACC. Transgenic rats and control Sprague Dawley rats, aged 6–8 weeks were kept under standard conditions with 12-h light–dark cycle and free access to water and food. The present study was carried out in accordance with the European Communities Council Directive of 24 November 1986 (86/609/EEC) for care of laboratory animals and after approval of the local government ethics committee. All efforts were made to minimize the number of animals used.

### SLICE PREPARATION

Animals were decapitated under deep ether anesthesia. The brain was rapidly taken out of the skull and immersed in ice-cold (1–4°C) sucrose-based solution (87 mM NaCl, 25 mM NaHCO_3,_ 10 mM D-glucose, 75 mM sucrose, 2.5 mM KCl, 1.25 mM NaH_2_PO_4_, 0.5 mM CaCl_2_, 7 mM MgCl_2_, bubbled with 95% O_2_ and 5% CO_2_, pH 7.4). Horizontal hippocampal slices (400 μm) were cut on a vibratome (Leica VT 1200S). Slices were kept at room temperature. For recordings, the slices were transferred to a submerged recording chamber. During the experiments, the chamber was perfused at a flow rate of 3 ml/min with oxygenated artificial cerebrospinal fluid (ACSF; 125 mM NaCl, 2.5 mM KCl, 1.25 mM NaH_2_PO_4_, 25 mM NaHCO_3_, 25 mM D-glucose, 2 mM CaCl_2_, 1 mM MgCl_2_, pH 7.4). All recordings were performed at 32°C.

### RECORDING OF EXTRACELLULAR FIELD POTENTIALS

Recording electrodes were pulled from borosilicate glass and filled with standard Artificial Cerebrospinal Fluid (ACSF, resistance 1–3 MΩ). Extracellular field potentials (FPs) were recorded from stratum radiatum (SR) of area CA1 every 60 s for at least 10 min (baseline recording) and then for at least 60 min following high- or low frequency stimulation (HFS or LFS). A concentric bipolar stimulation electrode was placed in SR of area CA1 to stimulate Schaffer collaterals and commissural fibers. FPs were recorded using a MultiClamp 700B amplifier, filtered at 3 kHz and sampled to a computer disk at 10 kHz using the Clampex 10.2 software (Molecular Devices, Sunnyvale, CA, USA). The stimulus intensity was set to evoke 30% of the maximum FP response in LTP, and 50% in LTD experiments (50–200 μA with stimulus duration of 100 μs). The HFS paradigm applied to SR consisted of a 1 s long stimulus train applied at frequencies of 100, 50, 30, or 10 Hz (pulse duration: 100 μs, each). For LFS trains 900 pulses were applied at 1 or 5 Hz (pulse duration: 100 μs, each; 4–6). FP recordings obtained from SR were analyzed by determining the slope of the ?eld excitatory postsynaptic potential (EPSP) between 10 and 90% of the peak amplitude. Summary graphs were prepared by normalizing all responses to the baseline obtained during 10 min prior to HFS or LFS and then averaged across experiments. Only slices with a stable baseline FP response were used for further analysis. All changes in long-term synaptic plasticity were evaluated by averaging responses at 50–60 min post-HFS or LFS and averaging across experiments. For metaplasticity experiments, the frequencies used were HFS 100 Hz for 1 s and LFS 1 Hz for 900 times for 15 min. After the first HFS or LFS for 15 min, LTP was measured for 60 min, then a second HFS or LFS for 15 min was applied, and LTP was measured for another 60 min. CNP (Calbiochem, Merck KGaA, Darmstadt, Germany) was prepared as stock solution, frozen, finally thawed on the day of the experiment and added to ACSF to reach the desired final concentration (100 nM).

### BEHAVIORAL TESTS

The temporal order to test the 11 wild type and the 12 transgenic rats were chosen randomly and changed for each experiment. All trials were recorded by a video camera and exploration time was measured by video analysis. The rats’ behavior was determined as exploration time when the animal approached closer than 2 cm to an object with its snout or forepaws (or both). After each trial, the arena and objects were cleaned carefully to avoid the presence of olfactory cues for the next rat. The available objects differed slightly in their shape, size and color. Before the first test – the novel object recognition task – rats were allowed to explore the arena during a habituation trial for 5 min, so that they could adapt to the new environment.

The object recognition task (ORT) was conducted in a square-shaped arena (100 cm × 100 cm), and used a similar approach as described in a previous report Goh and Manahan-Vaughan (2013). In the training trial (1st trial), two novel objects (i.e., A and B) were presented. Fifty minutes later (second trial) one familiar and one novel object (i.e., A and C) were presented. A further 24 h later (third trial) object A was presented with a new object (D). The objects were presented in the same location as the objects in the first trial. The objects and the recording chambers were cleaned thoroughly between task trials to ensure the absence of olfactory cues. The objects were distinctly different from one another and heavy so that they could not be moved by the rats.

In the SOR task the objects always remained the same (A and B), and the task was performed as previously described Goh and Manahan-Vaughan (2013). The position of object A remained constant, but in the second and third trial object B was placed in a distinctly new position in the chamber. As in the ORT experiments, the animals were allowed to explore the objects for 5 min. The second and third trials began 50 min and 24 h after the first trials, respectively.

In the open field locomotion test we examined motor function by means of measuring spontaneous activity in an open field (100 cm × 100 cm). Here, we differentiated between the entire running time of the animals and the time spent in the center of the area (without touching the walls).

### DATA ANALYSIS

Data were expressed as mean ± SEM. Statistical analysis was done with the aid of SigmaStat software (SPSS Inc., Chicago, IL, USA) performing Student’s *t*-test (unpaired), Mann–Whitney *U*-test or one-way analysis of variance (ANOVA) with repeated measures. The sample sizes (*n* values) for electrophysiological experiments indicate the number of slices from at least three different animals. Differences were considered significant with ^∗^*p* < 0.05 or ^∗∗^*p* < 0.01.

## RESULTS

### NPR-B-DEPENDENT cGMP SIGNALING IN NSE-NPR-BΔKC RATS

Transgenic rats overexpressing a dominant-negative NPR-B mutant under the control of a neuron-specific promoter (NSE-NPR-BΔKC; **Figure 1A**) were generated and identified by Southern blotting (**Figure 1B**). After breeding rats to homozygosity, tissue-specific expression of NSE-NPR-BΔKC in the brain was verified by RT-PCR. Other tissues, such as heart, lung, aorta and kidney do not express the transgene (**Figure 1C**). Western blot analysis showed that the NPR-B deletion construct was properly translated into a protein with the expected Mw of about 45 kDa in the transgenic animals but not in wild type rats (**Figure 1D**). To test whether expression of the dominant negative NSE-NPR-BΔKC mutant leads to the functional down-regulation of the NPR-B signaling, guanylyl cyclase assays in brain membrane preparations were performed (**Figures 1E,F**). The cGMP production of brain membranes derived from NSE-NPR-BΔKC rats stimulated with 10, 100, and 1000 nM ANP was comparable with those derived from wild-type rats, indicating that signaling via NPR-A was not affected in the transgene line (**Figure 1E**). In contrast, after stimulation with the same doses of CNP cGMP production significantly dampened in brain membrane preparations from NSE-NPR-BΔKC rats compared to wild-type rats. This result confirms the receptor-specific dominant-negative effect of the transgene expression on NPR-B signaling in the brain (**Figure 1F**).

### EFFECT OF NPR-B-DEPENDENT cGMP SIGNALING ON SYNAPTIC PLASTICITY IN HIPPOCAMPAL SLICES *IN VITRO*

**FIGURE 2:**
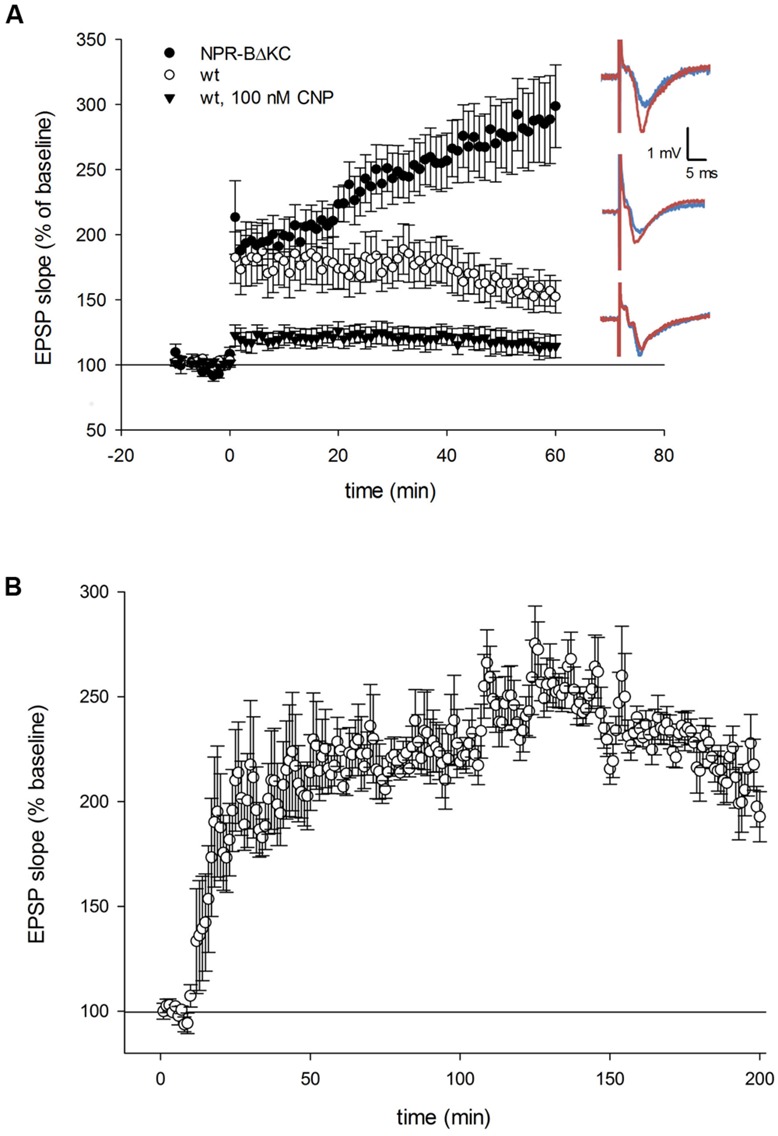
**(A)** Time course of the relative changes in the excitatory postsynaptic potential (EPSP) slope expressed in % of baseline stimulation induced by 100 Hz HFS for 1 s in hippocampal slices from NPR-BΔKC, from wild type rats (wt) and from wild type slices incubated with the NPR-B agonist CNP (100 nM). Sample traces of the electrically evoked EPSPs for the NPR-BΔKC, wild type slices, and wild type slices incubated with 100 nM CNP are shown on the right, and were recorded 10 min before and 60 min following high frequency stimulation (HFS, 100 Hz for 1 s). Note that LTP was more strongly expressed in slices from NPR-BΔKC rats (black circles) compared to wt (white circles). Bath application of 100 nM CNP (black triangles) caused attenuation of LTP. **(B)** The increase of the EPSP slope in NPR-BΔKC rats reaches saturation levels at 285% of baseline stimulation after 120 min. EPSPs decrease slowly to ∼200% of baseline stimulation after 180 min.

Here, we investigated the role of NPR-B and cGMP signaling in hippocampal synaptic plasticity. Field extracellular postsynaptic potentials (EPSP) were recorded from the SR of the CA1 by stimulating the Schaffer collaterals using high-frequency stimulation (HFS) at 100 Hz, which typically produces potentiation of the EPSP in the form of LTP. HFS resulted in LTP of 157 ± 12% 1 h after HFS was applied (*n* = 13) compared to baseline in wild-type (wt) rats (**Figure 2A**). In NPR-BΔKC rats, showing reduced CNP-dependent cGMP levels in brain membranes, HFS resulted in LTP that in its induction phase was similar to that seen in wt slices but after 60 min reached 285 ± 17% of baseline levels (*n* = 16, *p* < 0.01). On the opposite, application of 100 nM CNP to activate hippocampal NPR-B significantly inhibited LTP in wt rats (115 ± 10%, *n* = 7). Since the EPSP recordings in NPR-BΔKC rats did not reach saturation levels 60 min after HFS (**Figure 2A**), a longer time period of 200 min was recorded (**Figure 2B**). EPSP recordings showed a steep rise over the first 30 min, followed by a slower rise over the next 100 min and finally reached saturation levels at ∼250% of baseline values. This potentiation subsequently decreased somewhat to ∼200% of baseline values 180 min after HFS (**Figure 2B**).

### THE FREQUENCY-DEPENDENCE OF LTP AND LTD IS MODULATED BY NPR-B ACTIVATION THROUGH CNP AND FUNCTIONAL DOWN-REGULATION OF NPR-B SIGNALING

**FIGURE 3:**
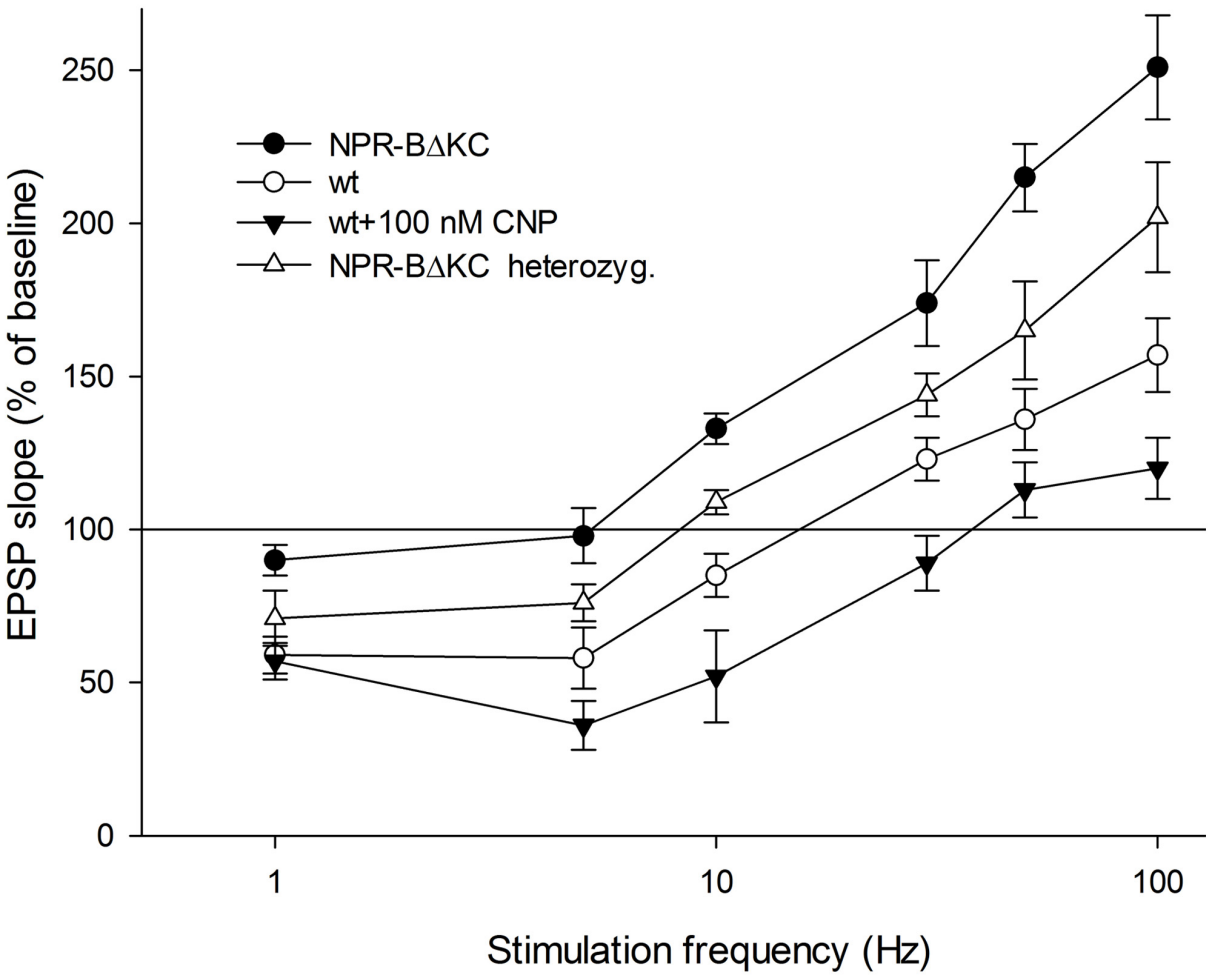
**Relative changes in the EPSP slope expressed in % of baseline stimulation after HF stimulation at 100 Hz for 1 s as recorded 50–60 min after stimulation in hippocampal slices from homozygous NPR-BΔKC rats, heterozygous NPR-BΔKC rats, slices from wild type rats (wt) and slices from wild type rats incubated with the NPR-B agonist CNP (100 nM).** Stimulation frequencies used were 1, 5, 10, 30, 50, and 100 Hz.

Next, we compared the frequency-dependence of synaptic plasticity in wt, homo- and heterozygous NPR-BΔKC animals. The extent of change of synaptic strength was assessed 60 min after application of afferent stimulation of Schaffer collateral-CA1 synapses (**Figure 3**). In wt animals LTD was elicited at frequencies of 1, 5, and 10 Hz stimulation. Stimulation at 30, 50, and 100 Hz led to potentiation of EPSP over baseline. In hetero- and homozygous NPR-BΔKC animals the frequency-dependence of synaptic plasticity was distinctly different to wt controls at frequencies of 5, 10, 30, 50, and 100 Hz (*p* < 0.01, *p* = 0.36 for 1 Hz stimulation, **Figure 3**). Thus, depending on gene dose the NPR-BΔKC mutation shifts LTP induction to lower stimulation frequencies compared to wt rats.

We then tested the effect of CNP application (100 nM) on synaptic plasticity in wt slices (**Figure 3**). Here, we observed that although LTD which was elicited using 1 Hz stimulation was equivalent to that seen in untreated wt controls, LTD induced by 5 Hz stimulation was significantly enhanced, and potent LTD was elicited by 10 Hz stimulation. In contrast, CNP-treatment resulted in a failure to induce LTP using 30 Hz stimulation. Stimulation at 50 and 100 Hz resulted in only a very small potentiation that was significantly less than that seen in untreated wt controls (*p* < 0.01). Taken together, these data suggest that under conditions of low cGMP levels the threshold for induction of LTP is lowered, whereas when cGMP is strongly elevated via the CNP/NPR-B/cGMP pathway the threshold for LTP induction is raised and LTD induction is facilitated.

### POSSIBLE MECHANISMS OF THE EFFECT OF FUNCTIONAL DOWN-REGULATION OF NPR-B SIGNALING ON LTP AND LTD IN HIPPOCAMPAL SLICES *IN VITRO*

**FIGURE 4:**
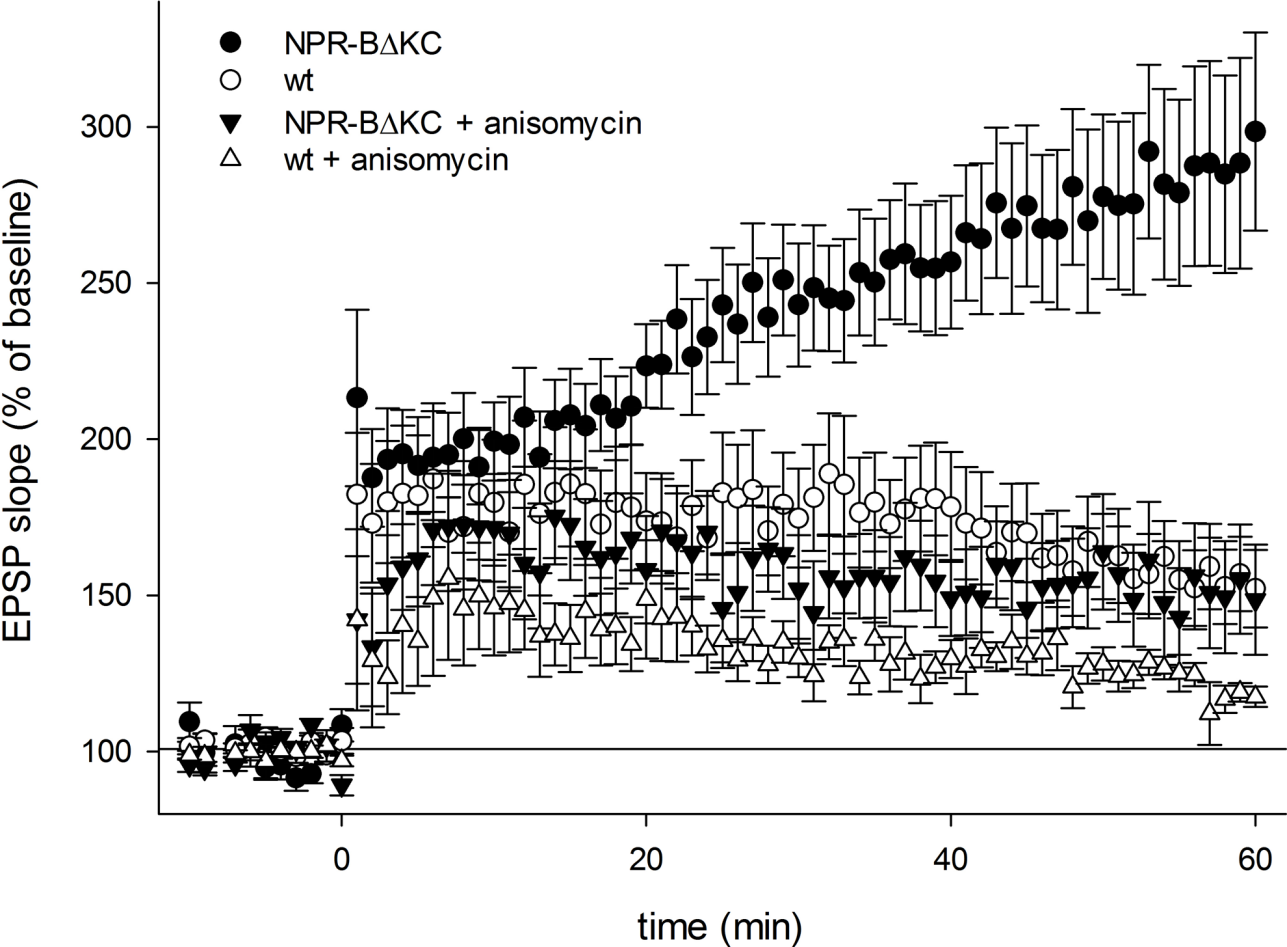
**Time course of the relative changes in the EPSP slope expressed in % of baseline stimulation induced by 100 Hz HFS for 1 s in hippocampal slices from NPR-BΔKC rats, wild type rats (wt), NPR-BΔKC rats incubated with anisomycin (20 μM) and wild type slices incubated with anisomycin (20 μM)**.

In search of possible molecular and cellular mechanisms responsible for the observed steady increase in EPSPs over a 60 min period following HFS, we repeated the experiments in the presence of anisomycin (20 μM), an inhibitor of protein synthesis (**Figure 4**). Anisomycin did not influence the induction of LTP but slightly reduced the potentiation of the EPSP signals in slices from wt rats, and reduced potentiation back to control levels in slices of NPR-BΔKC rats. These results indicate that protein synthesis is involved in the continuous increase of EPSP over a 60 min period in slices of NPR-BΔKC rats. However, the induction of LTP was unaffected by anisomycin in control and NPR-BΔKC rats.

**FIGURE 5:**
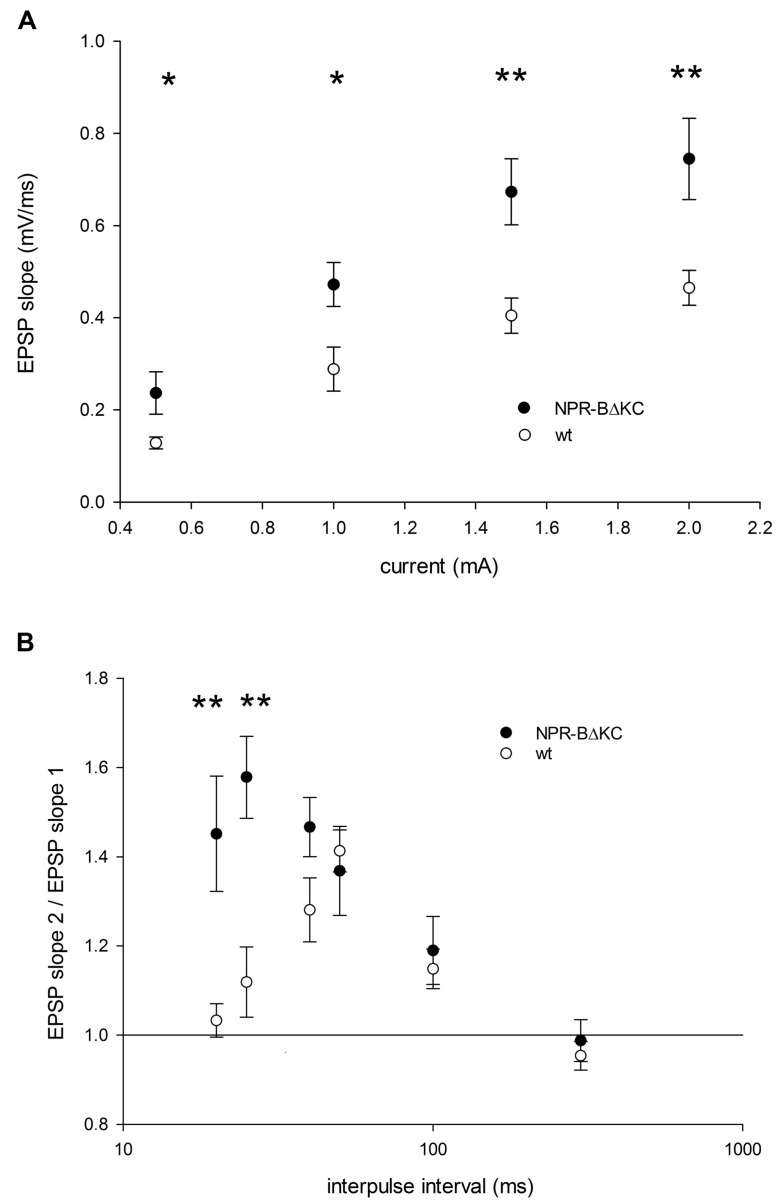
**(A)** EPSP slope as a function of stimulus current in slices of NPR-BΔKC (functional down-regulation of NPR-B signaling) and wild type (wt) rats. EPSP slopes recorded as mV/ms were determined between 0.5 and 2 mV. Note the greater excitability in hippocampal slices of NPR-BΔKC rats with significant differences at 0.5–2 mV stimulus current. **(B)** Paired-pulse facilitation as a function of interpulse interval current in slices of NPR-BΔKC (NPR-B) and wild type (wt) rats. Paired pulses as determined by the ratio of EPSP slope 2/EPSP slope 1 were recorded at 20, 25, 40, 50, 100, and 300 ms time intervals. Note the larger facilitation in slices of NPR-BΔKC rats at interpulse intervals of 20 and 25 ms. Asterisks indicate significant changes (*p < 0.05 and **p < 0.01).

We then tested whether NPR-BΔKC affects the intrinsic excitability in hippocampal neurons. Here we measured the EPSP in relation to the stimulation current (**Figure 5A**). We observed significantly higher responses in slices of NPR-BΔKC rats compared to wt using stimulation currents of 0.5–2 mA (*p* < 0.05). This increase in the input–output relationship indicates positive plastic changes in intrinsic excitability caused by reduced cGMP signaling that may underlie the observed lowering of LTP induction thresholds in NPR-BΔKC rats compared to wt rats.

Moreover, to test whether hippocampal inhibitory activity is affected in NPR-BΔKC rats we measured paired-pulse facilitation. Using different interpulse intervals (**Figure 5B**) we show larger facilitation in slices of NPR-BΔKC rats, particularly at the interpulse interval of 20 and 25 ms (*p* < 0.01), which is typically indicative of a loss of inhibitory GABAergic activity.

### THE EFFECT OF FUNCTIONAL DOWN-REGULATION OF NPR-B SIGNALING ON METAPLASTICITY IN HIPPOCAMPAL SLICES *IN VITRO*

**FIGURE 6:**
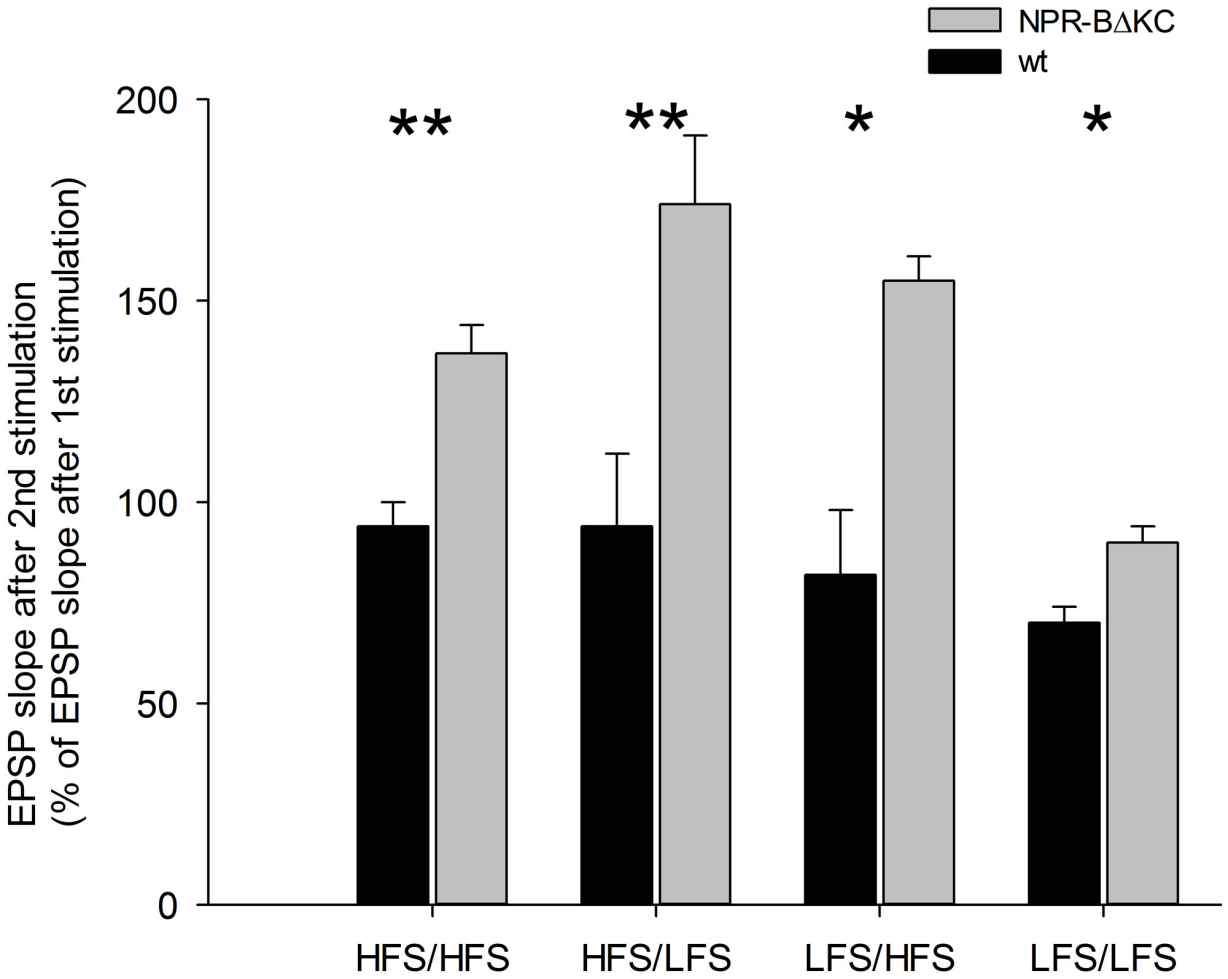
**Metaplasticity of EPSP when slices were stimulated a second time with HFS or LFS after an initial HFS, or with HFS or LFS after an initial LFS.** LFS,1Hz; HFS, 100 Hz. Bars represent mean values ± SEM of the relative changes in EPSP slope 50–60 min after the second stimulation in % of EPSP slope 50–60 min after the first stimulation. Note that LTP is significantly stronger expressed in slices from NPR-BΔKC rats in all cases. Asterisks indicate significant changes (*p < 0.05 and **p < 0.01).

Long-term potentiation and LTD depend on the current state of synapses as modulated by intrinsic but also extrinsic influences (Goh and Manahan-Vaughan, 2013). In other words, synaptic plasticity can be modified by activity including previous synaptic inhibition, the activity of modulatory afferents, and hormonal activity, for instance, of natriuretic peptide hormones. This plasticity of synaptic plasticity, also called metaplasticity, may play a role in some mechanisms of memory and learning (Dudek and Bear, 1993). Thus, we assessed effects of NPR-BΔKC on metaplasticity using repetitive HFS (100 Hz) or LFS (1 Hz) 60 min after an initial HFS (100 Hz) or LFS (1 Hz; **Figure 6**). Pretreatment with HFS did not increase LTP further following the second HFS in wild type rats, but increased LTP in NPR-BΔKC rats. Pretreatment with HFS abolished LTD in response to LFS in wt rats, but further increased LTP in NPR-BΔKC rats. Similarly, LFS pretreatment further increased LTP following the second HFS in NPR-BΔKC rats, but had no effect in the LFS/LFS combination. Thus, in all combinations of LFS and HFS a significant bigger potentiation was observed after the second stimulation (% of potentiation 60 min after first stimulation) in NPR-BΔKC rats compared to wt rats (*p* < 0.05). These results indicate that the effect of the NPR-B/cGMP signaling system on LTP and LTD also affects metaplasticity.

### MEASURING ANXIETY/CURIOSITY AND LEARNING AND MEMORY IN TRANSGENIC RATS EXPRESSING DOMINANT-NEGATIVE NPR-BΔKC

**FIGURE 7:**
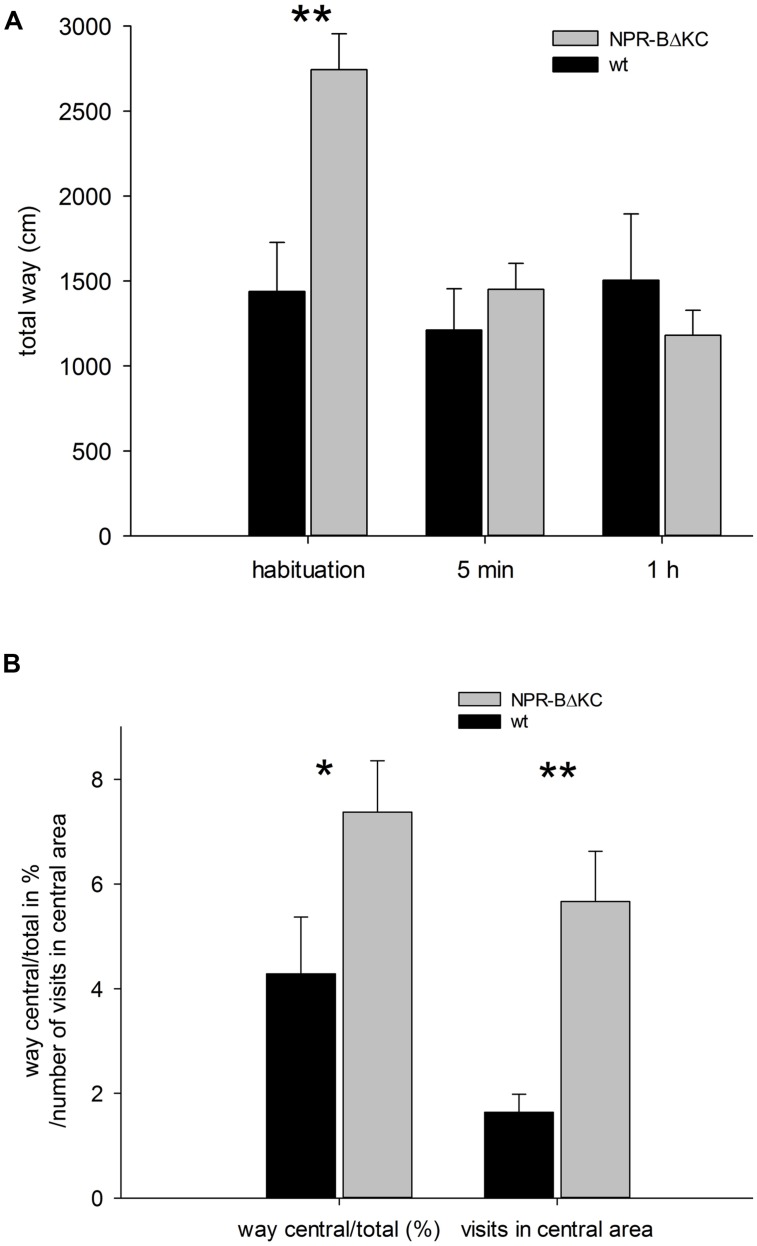
**In the open field test NPR-BΔKC and wild type rats (wt) were placed in an open field arena and movement of animals was observed to examine exploratory behavior and anxiety. (A)** NPR-BΔKC rats moved more (total way in cm) during initial habituation. In the second trial after 5 min and the third trial after 1 h the rats from both groups did not show significant differences in the total distance moved. **(B)** NPR-BΔKC rats showed a higher ratio of movement in the central area versus total movement (way central/total in %). Transgenic animals also demonstrated an increase in the number of visits in the central area as compared to wt animals. Asterisks indicate significant changes (*p < 0.05 and **p < 0.01).

To evaluate changes in learning behavior of NPR-BΔKC animals in behavioral experiments, and relate these results with effects of NPR-BΔKC on synaptic plasticity, we performed open field, novel object recognition and SOR tests. In the open field test, anxiety and curiosity were compared between NPR-BΔKC and control rats. We observed significant differences in locomotion of NPR-BΔKC rats in the first trial in the arena (habituation; **Figure 7A**, *p* < 0.01). The greater willingness of NPR-BΔKC rats to run can be understood as a sign of greater curiosity and reduced anxiety. However, in the second and third trial after 5 min and 1 h, respectively, the rats from both groups did not show significant differences in the total distance moved. On the other hand, the NPR-BΔKC rats moved significantly longer distances in the central area of the arena compared to the total distance traveled (**Figure 7B**, *p* < 0.05). NPR-BΔKC rats also made significantly more visits to the central area (**Figure 7B**, *p* < 0.01). These results indicate that NPR-BΔKC rats show reduced levels of anxiety leading to enhanced exploratory behavior.

**FIGURE 8:**
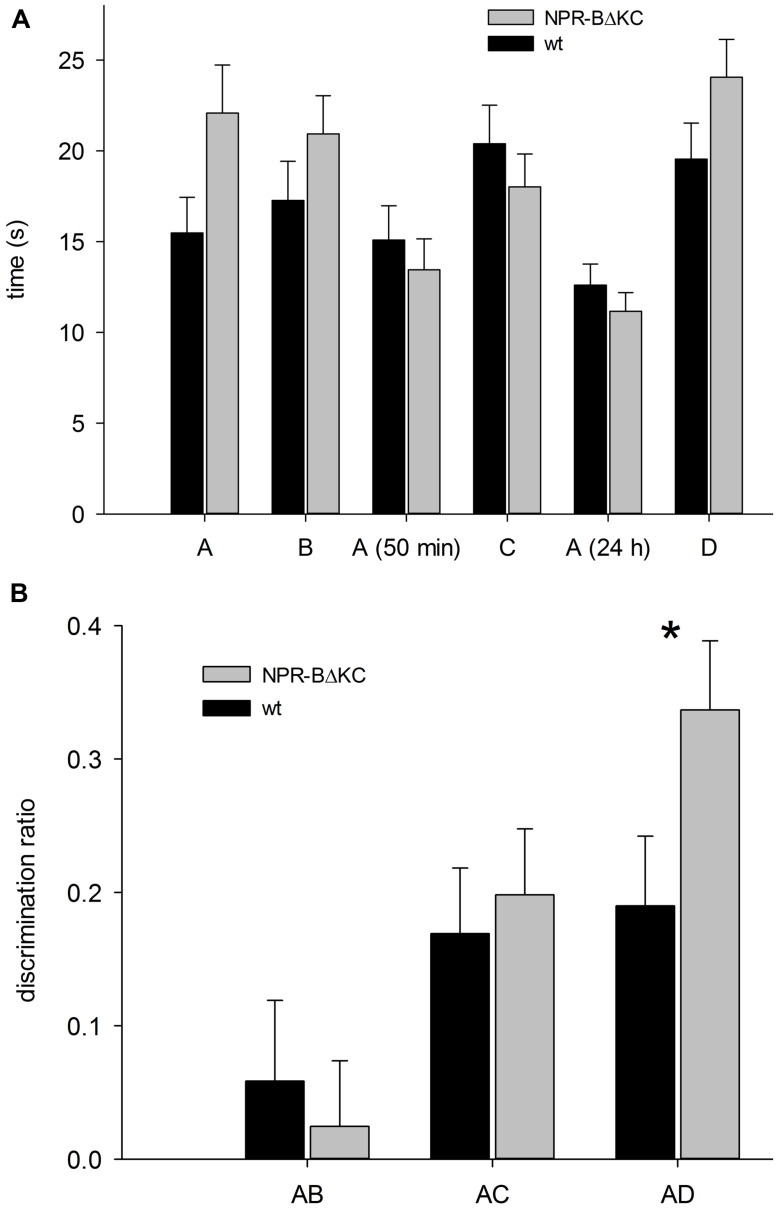
**In the novel ORT NPR-BΔKC rats and wild type rats (wt) were placed in an open field arena with novel and familiar objects. (A)** NPR-BΔKC rats revealed higher exploratory activity when different objects (A and B) were presented as measured by the time spent in the vicinity to the objects (time in s). Exposure to the familiar (A) or a novel object after 50 min (C) or 24 h (D) demonstrated that NPR-BΔKC rats had an increased preference for novel object D after 24 h compared to wt animals. **(B)** Transgenic animals showed a significantly greater preference for the novel object compared to wt controls as analyzed by the discrimination ratio of exploration between new and old object after 24 h (AD) compared to wt control animals. Asterisks indicate significant changes (*p < 0.05).

**FIGURE 9:**
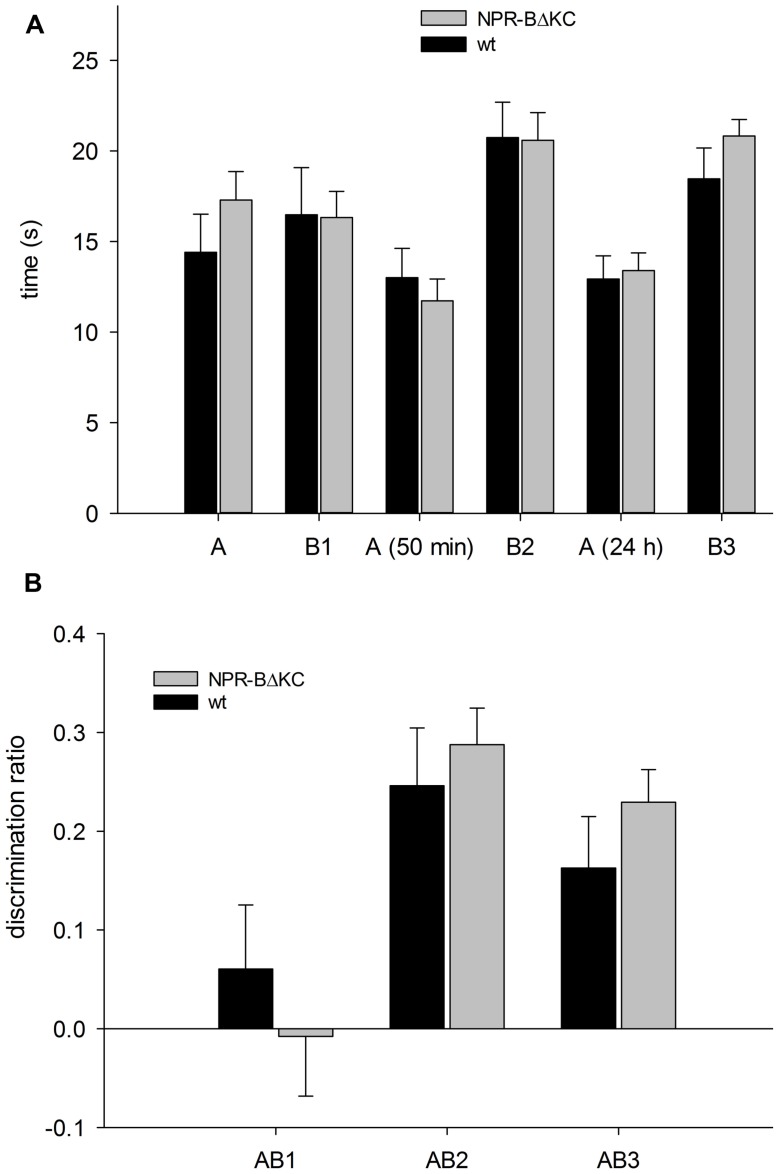
**In the spatial object recognition test NPR-BΔKC and wild type rats (wt) were placed in an open field arena with two objects (A and B1, B2, or B3), and with objects B2 and B3 moved to a different place after 50 min and 24 h, respectively. (A)** Both, NPR-BΔKC and wild type rats (wt) showed enhanced interest for the object in a novel place after 50 min (B2) and 24 h (B3). **(B)** The calculation of the discrimination ratio calculation reveals no significant differences in the interest for the object in a novel place after 50 min (B2) and 24 h (B3) between NPR-BΔKC and wild type rats (wt).

In the novel object recognition test (ORT), upon exposure to the familiar object (A) and the unfamiliar object (C at 50 min, D at 24 h), one would expect that the animals show a markedly greater interest in the unfamiliar objects compared to object A (22). This was the case both for wt and transgenic animals (**Figure 8A**). The analysis also revealed a significantly higher exploratory activity for NPR-BΔKC rats in the initial 5 min when objects (A and B) were introduced into the arena (**Figure 8A**, *p* = 0.065). Object discrimination ratios were calculated to verify that the animals showed a preference of the novel vs. the familiar object (**Figure 8B**). No significantly different discrimination ratios were recorded in the first trial suggesting that the animals found both novel objects equally interesting. Upon exposure to object A and novel object C in the second trial, a significant preference for object C over object A was seen in both animal groups. A preference for the novel object was still evident 24 h after first object exposure, whereby the transgenic animals showed a significantly greater preference for object D compared to wt controls (*p* < 0.05). This indicates that long term memory is facilitated in NPR-BΔKC rats. In the SOR test, the two groups exhibited the expected learning performance, but no significant differences between the two groups were observed (**Figure 9A**). In the first trial AB1, the second trial after 50 min. and the third trial after 24 h NPR-BΔKC rats did not exhibited a significantly changed discrimination ratio (**Figure 9B**). Although NPR-BΔKC rats showed a tendency for an increased discrimination ratio AB3 in the third trial (**Figure 9B**), SOR was not significantly different between NPR-BΔKC rats and wt animals.

## DISCUSSION

The second messenger cGMP modulates synaptic transmission and learning processes. This has been attributed mainly to calcium- and nitric oxide (NO)-dependent activation of sGCs (Schuman and Madison, 1991; Arancio et al., 1996; Son et al., 1998). However, other cGMP-producing enzymes including the transmembrane guanylyl cyclase NPR-B are expressed in the brain, including the hippocampal formation. NPR-B is activated by the CNP and produces cGMP in hippocampal neurons (Brackmann et al., 2005). CNP and its receptor NPR-B are strongly expressed in hippocampal regions CA1-3 (Langub et al., 1995; Herman et al., 1996), which are well-described model systems for studying synaptic plasticity and metaplasticity and their relation to learning and memory (Dudek and Bear, 1993; Manahan-Vaughan and Braunewell, 1999; Braunewell and Manahan-Vaughan, 2001). Evidence that the CNP/NPR-B system is able to modulate synaptic transmission came from our recent study showing that application of CNP, the ligand for NPR-B, negatively affects LTP, but positively affects LTD in hippocampal slices (Decker et al., 2008, 2009, 2010). Our present results indicate that transgenic rats expressing a dominant-negative mutant (NPR-BΔKC) of NPR-B in the brain, display changes of bidirectional plasticity in the opposite way. Rats expressing the NPR-BΔKC mutant, which specifically inhibits cGMP production of NPR-B without affecting NPR-A, showed enhanced LTP and reduced LTD induction. When frequency-dependence of synaptic modification in the range of 1–100 Hz was assessed transgenic rats expressed LTP at lower stimulation frequencies than wild-type controls and at the same time exhibited enhancements in exploratory and learning behavior. These results indicate that modulation of bidirectional plasticity via NPR-B-dependent cGMP signaling is related to learning and memory behavior in rats, and are in line with the notion that NPR-B and the associated cGMP signaling pathway constitutes a negative feed forward or feedback loop regulating synaptic plasticity.

It is worth mentioning that exploratory activity and spatial learning are associated with LTP and LTD. In rats, exploratory behavior of a new environment containing unfamiliar objects and/or familiar objects in new locations facilitated LTD and impaired LTP, whereas exploration of the new environment itself, in the absence of objects, impaired LTD and facilitated LTP (Manahan-Vaughan and Braunewell, 1999; Braunewell and Manahan-Vaughan, 2001; Kemp and Manahan-Vaughan, 2004, 2007, 2008). More recently, it was shown in mice that object recognition facilitates hippocampal LTD and impairs LTP, indicating that both forms of plasticity work in concert in the creation of spatial memory. However, LTP and LTD might contribute to different components of a spatial representation and learning (Goh and Manahan-Vaughan, 2013). In NPR-BΔKC mutant rats, showing enhanced LTP and reduced LTD, we found improved object recognition. Although our experiments did not investigate the exact contributions of LTP and LTD to SOR, these results further substantiate the link between LTP and LTD as cellular mechanism required for spatial memory tasks (Goh and Manahan-Vaughan, 2013), and link cGMP signaling via NPR-B with synaptic plasticity and learning.

In regard to the possible molecular and cellular mechanisms of how NPR-B affects synaptic plasticity in the hippocampal network, our results indicate that protein synthesis is involved in the observed effect on EPSPs. The late onset but continuous increase of EPSPs over a prolonged time period in NPR-BΔKC mutant rats was sensitive to the protein synthesis inhibitor anisomycin, indicating that the link between reduced cGMP production and enhanced LTP involves protein synthesis. Moreover, the increase in the input-output relationship of LTP indicates positive alterations in the intrinsic excitability of neurons probably leading to the shift in LTP induction frequencies. This is most likely a sign of loss of inhibitory GABAergic activity in the slices of NPR-BΔKC mutant rats. This assumption is in agreement with our previous results showing that CNP activation of NPR-B in hippocampal slices leads to inhibition of LTP by modulating GABA(A)-mediated inhibition of the hippocampal network (Decker et al., 2008, 2009, 2010). Similarly, the effect of the inhibition of NPR-B-dependent cyclase activity on metaplasticity also indicates that there is a loss of negative feedback in the hippocampus of transgenic rats. In our experiments all combinations of LFS and HFS, with the exception of the LFS/LFS combination, led to a significant increase in LTP after the second stimulation in NPR-BΔKC rats. These differences in metaplasticity are most likely explained by the fact that 1 Hz LFS did not induce LTD in the transgenic rats, and that LTP may not have been saturated at 1 h and typically reached much higher levels than in wt animals. These differences likely contribute to the observed changes in metaplasticity in transgenic animals. Thus, the NPR-B/cGMP signaling system may play a role in metaplasticity, for instance as one of the regulatory feedback mechanism, to link previous experience or the hormonal state of the organism to synaptic plasticity.

In our previous studies we have postulated that the physiological significance of the CNP/NPR-B signaling system in the hippocampus may be to restrict synaptic plasticity and possibly learning and memory behaviors in stress-linked situations and thus link anxiogenic and limbic functions. This assumption was based on preclinical studies in rats and human studies which have shown that natriuretic peptides differentially modulate endocrine and behavioral stress responses (Kellner et al., 2003). CNP enhances the release of ACTH and cortisol (Kellner et al., 2003) and had anxiogenic effects in rats and in humans (Jahn et al., 2001). We have previously shown that exposure of rat strains to stress, in form of a novel open platform environment, induced a significant elevation in serum corticosterone levels but did not facilitate LTD expression (Manahan-Vaughan and Braunewell, 1999). However, different or longer lasting forms of stress activating the NPR-B system are likely to modulate bidirectional plasticity and thus lead to reduced learning and memory-related behavior. Interestingly, in the transgenic rats showing reduction of the NPR-B and cGMP signaling system we observe reduced anxiety levels which are observed as an enhanced exploratory behavior in the open field test as well as in object recognition tests. Enhanced exploratory and novelty seeking behavior is likely linked to learning and memory behavior (Manahan-Vaughan and Braunewell, 1999; Braunewell and Manahan-Vaughan, 2001). Although we have seen significant differences in learning behavior only in novel object recognition and not in the SOR test, enhanced exploratory behavior was noticed in open field and object recognition tests alike. More detailed behavioral analysis will be necessary to further characterize the role of NPR-B for distinct learning and memory paradigms.

Interestingly, GC-C another member of the family of transmembrane guanylyl cyclases (Potter et al., 2009; Potter, 2011), previously thought to be expressed mainly in neurons of the intestine, was identified in midbrain dopaminergic neurons, particularly in midbrain ventral tegmental area and substantia nigra compacta (VTA/SNc) neurons that regulate many important behavioral processes (Gong et al., 2011). Dysfunction of these neurons is associated with attention deficit hyperactivity disorder (ADHD) and schizophrenia. Mice in which GC-C has been knocked out exhibit hyperactivity and attention deficits which resemble behavioral symptoms of ADHD (Gong et al., 2011). Similarly, the functional down regulation of NPR-B in NPR-BΔKC rats showed increased activity and exploratory behavior. Thus, dysfunction of receptor guanylyl cyclases and the cGMP-pathway may be involved in a variety of additional CNS disorders, for instance deficits in cGMP signaling pathways and neuroplasticity are hypothesized to underlie the pathophysiology of major depressive disorder (MDD; Reierson et al., 2011). This is in line with the hypothesis that increased cGMP signaling in the brain restricts excitability of neurons, and thereby controls a range of behaviors including hyperactivity, anxiety, attention, learning and memory.

## Conflict of Interest Statement

The authors declare that the research was conducted in the absence of any commercial or financial relationships that could be construed as a potential conflict of interest.
